# Cold acclimation causes fiber type-specific responses in glucose and fat metabolism in rat skeletal muscles

**DOI:** 10.1038/s41598-017-15842-3

**Published:** 2017-11-13

**Authors:** Diane M. Sepa-Kishi, Yass Sotoudeh-Nia, Ayesha Iqbal, George Bikopoulos, Rolando B. Ceddia

**Affiliations:** Muscle Health Research Center, School of Kinesiology and Health Science, York University, Toronto, Ontario Canada

## Abstract

This study investigated fiber type-specific metabolic responses and the molecular mechanisms that regulate glucose and fat metabolism in oxidative and glycolytic muscles upon cold acclimation. Male Wistar rats were exposed to cold (4 °C) for 7 days, and then glycogen synthesis and content, glucose and palmitate oxidation, and the molecular mechanisms underlying these metabolic pathways were assessed in soleus (Sol), extensor digitorum longus (EDL), and epitrochlearis (Epit) muscles. Cold acclimation increased glycogen synthesis, glycogen content, glucose oxidation, and reduced glycogen synthase (GS) phosphorylation only in Sol muscles. Protein kinase B (AKT), glycogen synthase kinase 3 (GSK3), and AMP-activated protein kinase (AMPK) phosphorylation increased in all three muscles upon cold acclimation. Cold acclimation increased palmitate oxidation, gene expression of the transcriptional co-activator *Pgc-1α*, lipoprotein lipase (*Lpl*), fatty acid transporter (*Cd36)*, and Sarco/endoplasmic reticulum Ca^2+^-ATPase (*Serca*) in Sol, EDL, and Epit muscles. Sarcolipin was only detected and had its content increased in Sol muscles. In conclusion, cold-induced thermogenesis activated similar signaling pathways in oxidative and glycolytic muscles, but the metabolic fate of glucose differed in skeletal muscles with distinct fiber type composition. Furthermore, only muscles rich in type I fibers appeared to have the capacity for sarcolipin-mediated SERCA uncoupling.

## Introduction

Acute cold exposure and cold acclimation in rodents have been reported to markedly increase whole-body energy expenditure^1^, which is accompanied by profound metabolic changes including hyperphagia, reduced insulinemia, enhanced hepatic glucose production, and increased glucose and fat utilization by peripheral tissues^1–6^. The brown adipose tissue (BAT) is the main site of non-shivering thermogenesis^7–9^ and greatly contributes to the metabolic changes induced by cold exposure^1^. However, skeletal muscles also have the capacity for both shivering and non-shivering thermogenesis^4,10^, and have been demonstrated to significantly increase substrate utilization under acute cold exposure^2^ and cold acclimating conditions^4^. Skeletal muscles make up a large proportion of total body mass (~40% and 30% in men and women, respectively)^11^ and, therefore, can play an important role in determining the metabolic adaptive responses to cold-induced thermogenesis. From a metabolic perspective, fatty acids are considered the principal fuel oxidized during cold-induced thermogenesis^6^; however, fatty acids cannot entirely substitute for glucose as a fuel for cold-induced thermogenesis. In fact, glucose appears to play an important regulatory role by generating essential glycolytic metabolites or by replenishing citric acid cycle intermediates to sustain an elevated rate of fatty acid oxidation (FAO) under conditions of cold exposure^12,13^. This could be particularly relevant in highly oxidative muscles that consume large amounts of fatty acids to fuel cold-induced thermogenesis.

Because cold exposure leads to insulinopenia^4,12,14^, it has been suggested that the cold-induced increase in glucose uptake in skeletal muscles is primarily diverted to fuel the oxidative pathway that is upregulated in an insulin-independent manner^12^. This can be attributed to cold-induced shivering (contractile activity)^14–16^, since it resembles the effects of exercise on glucose uptake in this tissue^14,17^. However, shivering progressively stops and non-shivering thermogenesis takes over as cold acclimation takes place^18,19^. Despite the cessation of shivering, glucose and fat metabolism remain elevated in skeletal muscles of cold acclimated rats^1,4^, suggesting that mechanisms independent of contractile activity operate to maintain elevated rates of substrate utilization in skeletal muscles in these animals. In this context, it has been hypothesized that prolonged cold exposure increases glucose uptake and its metabolism in skeletal muscles by: (a) increasing tissue sensitivity to insulin, and (b) stimulating glucose uptake via insulin-independent pathways^4,14^. These effects have been attributed to elevated release of norepinephrine from sympathetic nerve endings as a consequence of sympathetic nervous system (SNS) activation by cold^4^. However, the molecular mechanisms by which cold-induced thermogenesis influences the activity of the glucose transport protein and glucose and fat metabolism in skeletal muscles remain largely undetermined.

More recently, it has been demonstrated that elevated sarco/endoplasmic reticulum Ca^2+^-ATPase (SERCA) activity plays an important role in non-shivering thermogenesis in skeletal muscles^10^. As an ATP-driven pump, SERCA efficiently couples the hydrolysis of ATP to the transport of Ca^2+^ across the membrane, and it does so in a way that two Ca^2+^ ions are transported for each ATP molecule hydrolysed^20,21^. The mechanism by which cold increases SERCA-mediated non-shivering thermogenesis has been attributed to the presence of sarcolipin (SLN), a protein that uncouples hydrolysis of ATP from the transport of Ca^2+^ by SERCA^10,21,22^. However, the different SERCA isoforms and SLN do not seem to be equally expressed in oxidative and glycolytic muscles^10,23,24^, which suggests that heterogeneity exists with respect to the contribution of SLN-mediated SERCA uncoupling to cold-induced enhancement of glucose and fat metabolism among muscles of different fiber type composition. Another potential mechanism by which skeletal muscles could enhance substrate utilization in an insulin-independent manner is through the activation of the cellular energy sensor AMP-activated protein kinase (AMPK). Activation of AMPK could be initially triggered by muscle contractions (shivering) and ATP consumption and remain activated as non-shivering thermogenesis takes over due to SERCA uncoupling or even increased activity of the Na^+^-K^+^ ATPase under the influence of SNS activity and norepinephrine release. In its activated state, AMPK is well known for promoting insulin-independent glucose uptake and enhancement of FAO^25^ in skeletal muscle cells. Besides responding to alterations in the intracellular AMP:ATP ratio, AMPK can also be covalently activated by calcium/calmodulin dependent kinase kinase 2 (CAMKK2) in response to alterations in intracellular Ca^2+^ flux^25^. Therefore, AMPK activity could be induced by SERCA uncoupling in skeletal muscle under conditions of cold acclimation. However, the expression of the α catalytic and β regulatory subunits of AMPK differs between oxidative and glycolytic muscle fibers^26–28^, suggesting that the potential role of AMPK in the regulation of glucose and fatty acid metabolism under cold acclimation and non-shivering thermogenesis is also fibre type-specific. Because the molecular mechanisms that regulate energy metabolism in skeletal muscles operate in a fiber-type dependent manner, we hypothesized that substrate partitioning in skeletal muscles would be distinctly regulated in oxidative and glycolytic muscles under conditions of cold-induced thermogenesis. In order to test this hypothesis, we exposed rats to a cold acclimation protocol and then assessed glucose and fatty acid metabolism in skeletal muscles with distinct fiber type distribution. We also investigated the insulin-dependent and independent signaling pathways that drive substrate partitioning and assessed the molecular mechanisms underlying the adaptive metabolic responses of oxidative and glycolytic muscles to cold acclimation.

## Results

### Effects of cold exposure on food intake, body weight, and circulating glucose, insulin and non-esterified fatty acids (NEFAs)

Food intake was 45% higher in cold-acclimated rats compared to controls. Despite hyperphagia, no significant differences were found in body weight, glycemia, and NEFAs at the end of the cold exposure protocol between control and cold-acclimated rats (data not shown). In contrast, circulating insulin levels were reduced by ~35% after 1 day of cold exposure (control 0.288 ± 0.03 nM vs. cold 0.188 ± 0.01 nM) and remained significantly lower throughout the entire 7-day cold-acclimation period.

### Effects of cold exposure on basal and insulin-stimulated skeletal muscle glycogen synthesis and glucose oxidation

**Figure 1:**
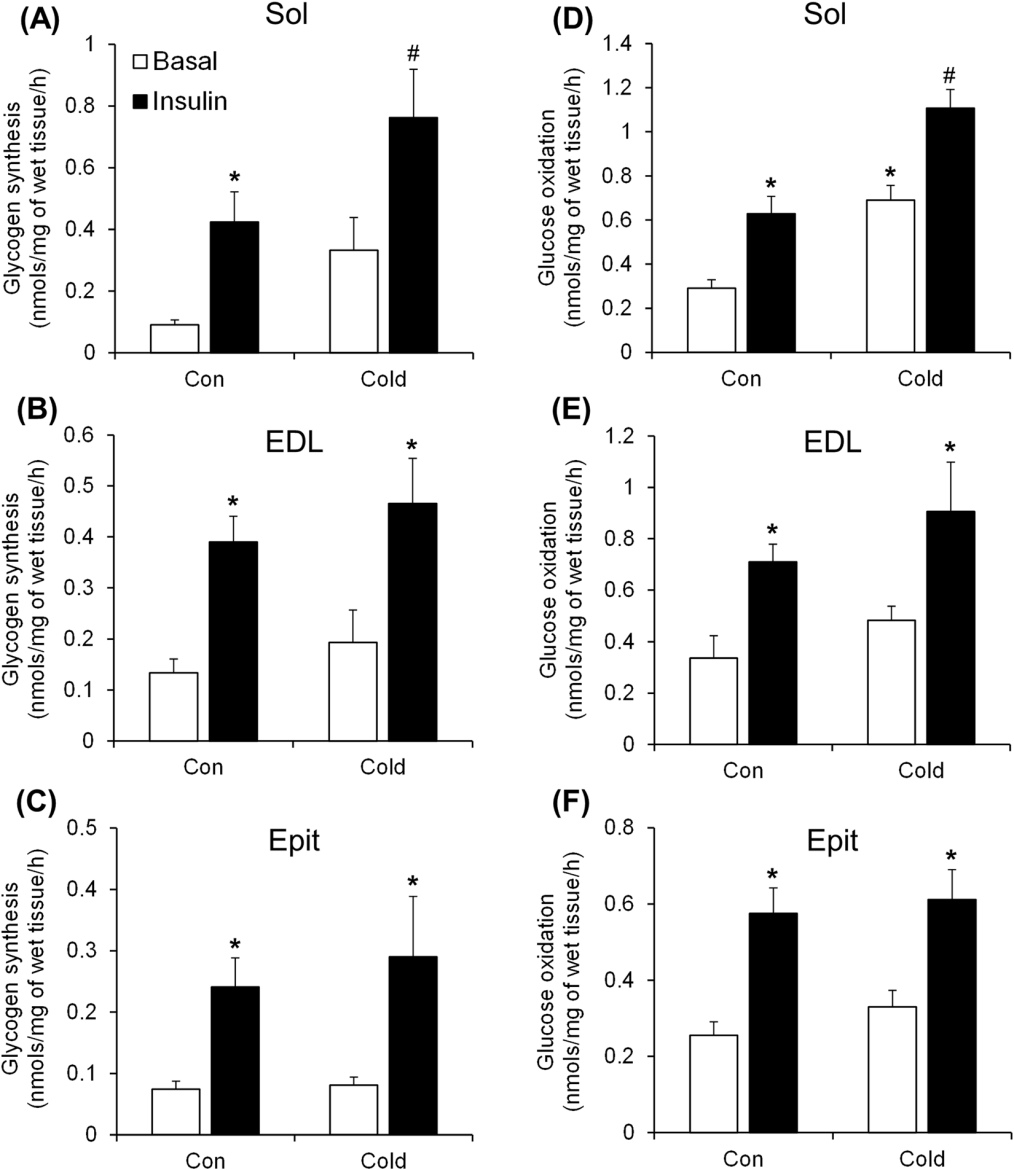
Cold exposure increases insulin-stimulated glycogen synthesis (**A**) and basal and insulin-stimulated glucose oxidation (**D**) in the Sol, but does not alter glycogen synthesis or glucose oxidation in the EDL (**B** and **E**) or the Epit (**C** and **F**). Two-way ANOVA, n = 11. *Indicates main effect of insulin (p < 0.05 vs. Basal) or main effect of cold exposure (p < 0.05 vs. Control (Con) Basal). ^#^Indicates interaction between insulin and cold exposure (p < 0.05 vs. all other conditions).

Under basal conditions, the rates of glycogen synthesis were not significantly altered by cold exposure in all three muscles (Fig. 1A–C), although a trend of increase was found for the Sol muscle (Fig. 1A). As expected, Sol, EDL, and Epit muscles from control rats elicited 4.7-fold (Fig. 1A), 2.9-fold (Fig. 1B), and 3.4-fold (Fig. 1C) increases in insulin-stimulated glycogen synthesis, respectively. Similarly, in muscles from cold-exposed rats, insulin significantly increased glycogen synthesis by 2.3-fold in the Sol (Fig. 1A), 2.4-fold in the EDL (Fig. 1B), and 3.6-fold in the Epit muscle (Fig. 1C). However, in the Sol muscles we found that cold exposure had an additive effect, which enhanced insulin-stimulated glycogen synthesis by 80% when compared to control rats (Fig. 1A). Glucose oxidation in Sol, EDL, and Epit muscles from control rats also increased by ~2.2-fold (Fig. 1D), 2.1-fold (Fig. 1E), and 2.25-fold (Fig. 1F), respectively, when stimulated by insulin. Similarly to glycogen synthesis, all three muscles increased their rates of glucose oxidation when stimulated by insulin (Fig. 1D–F). However, an additive effect (~76%) in glucose oxidation was only observed in the Sol muscle (Fig. 1D) when compared to controls.

### Effects of cold exposure on skeletal muscle glycogen content under basal and insulin-stimulated conditions

**Figure 2:**
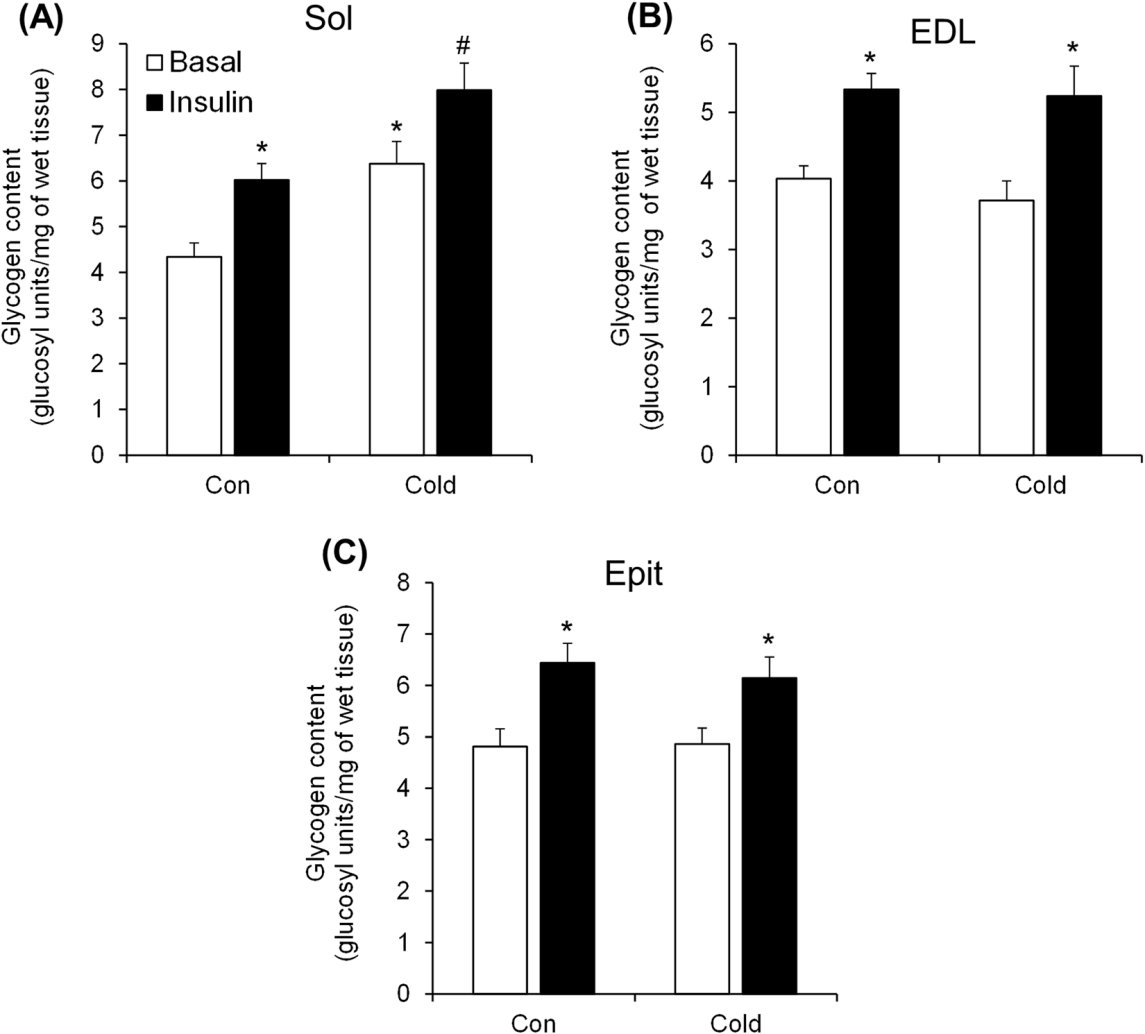
Cold exposure increases basal and insulin-stimulated glycogen content in the Sol (**A**), but not in the EDL (**B**) or Epit (**C**). Two-way ANOVA, n = 12. *Indicates main effect of insulin (p < 0.05 vs. Basal) or main effect of cold exposure (p < 0.05 vs. Control (Con) Basal). ^#^Indicates interaction between insulin and cold exposure (p < 0.05 vs. all other conditions).

Sol, EDL, and Epit muscles from control rats had similar contents of glycogen (Fig. 2A–C), and upon stimulation with insulin it increased by 39% (Fig. 2A), 32% (Fig. 2B), and 34% (Fig. 2C), respectively. In muscles from cold-exposed rats, insulin stimulation increased glycogen content by 22% in the Sol, by 41% in the EDL, and by 26% in the Epit muscles. However, only the Sol muscle displayed an additive effect of ~50% and 30% under basal and insulin-stimulated conditions (Fig. 2A), respectively, when compared to control. This additive effect of cold exposure on glycogen content is consistent with similar increases in glycogen synthesis observed only in the Sol muscle.

### Effects of cold exposure on the phosphorylation of AKT, GSK3, and GS, and on Glut4 and Glut1 gene expression in skeletal muscles

**Figure 3:**
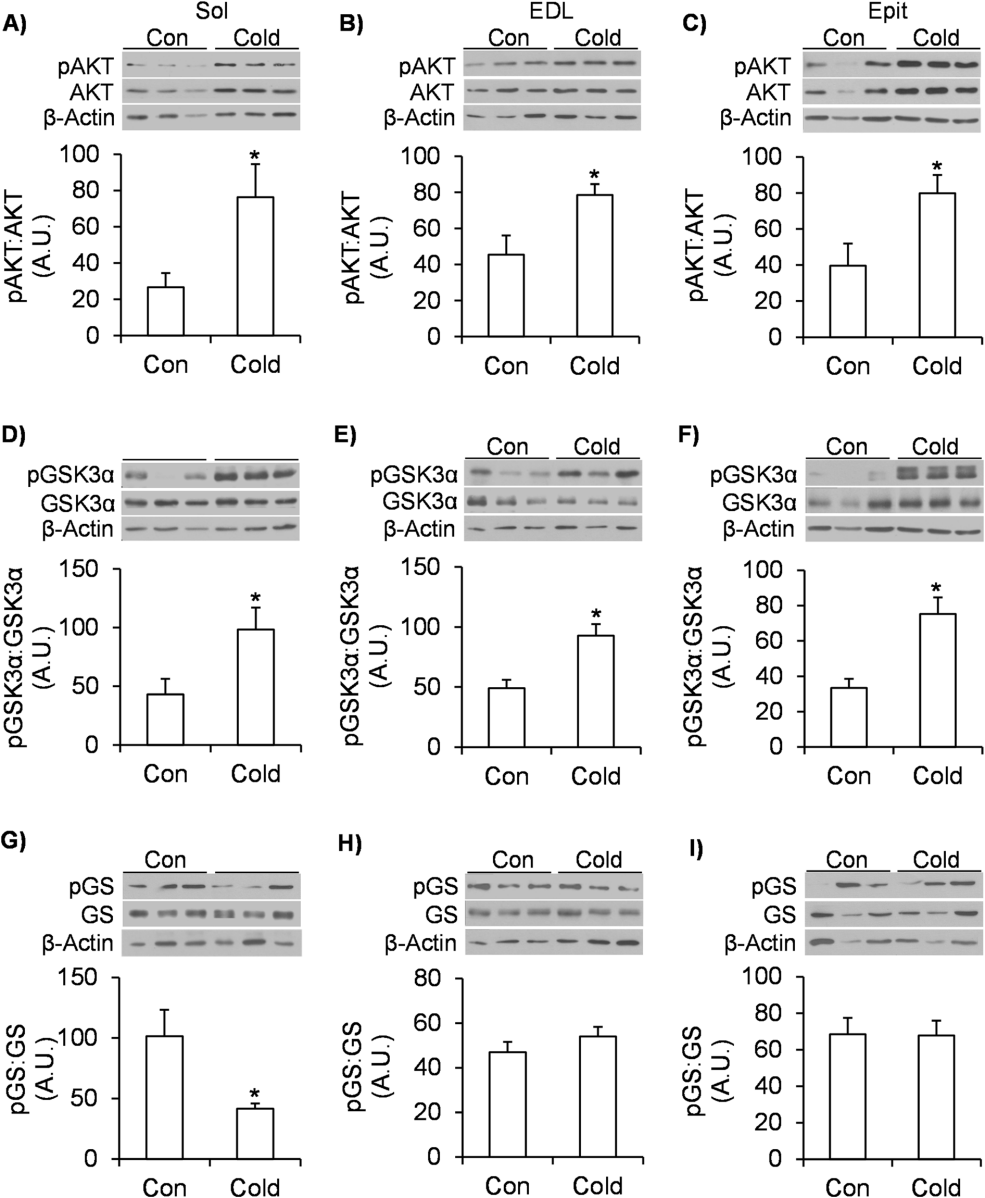
Cold acclimation increases AKT (**A** to **C**) and GSK3 (**D** to **F**) phosphorylation in Sol, EDL and Epit muscles, but it only decreases GS phosphorylation (**G** to **I**) in the Sol muscle. Age- and weight-matched animals were either kept at room temperature or cold-exposed (4 °C) for 7 days. Following the protocol, a portion of each skeletal muscle (that was not incubated) was extracted, flash frozen in liquid nitrogen and stored at −80 °C until western blot analysis. Student’s t-test, n = 6–12. *p < 0.05 vs. Control (Con).

**Figure 4:**
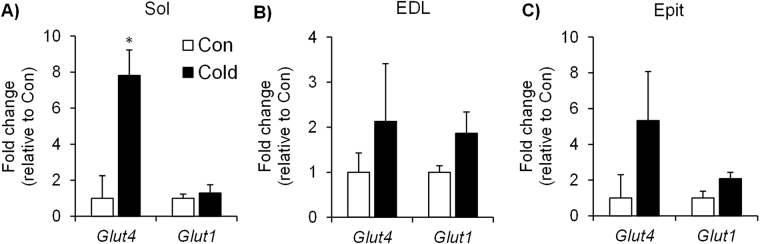
Cold exposure increases Glut4 expression in Sol muscles (**A**), but does not significantly change the gene expression of *Glut1* in Sol, nor of *Glut1* and *Glut4* in EDL (**B**) and Epit (**C**). Age- and weight-matched animals were either kept at room temperature or cold-exposed (4 °C) for 7 days. Following the protocol, a portion of each skeletal muscle (that was not incubated) was extracted, flash frozen in liquid nitrogen and stored at −80 °C until PCR analysis. Student’s t-test, n = 6–9. *p < 0.05 vs. Control (Con).

Cold exposure significantly increased the phosphorylation of AKT by 2.9-fold (Fig. 3A), 1.73-fold (Fig. 3B), and 2-fold (Fig. 3C) in Sol, EDL, and Epit muscles, respectively. A similar effect was observed for GSK3α phosphorylation that significantly increased by ~2.3-fold in the Sol (Fig. 3D), by 1.9-fold in the EDL (Fig. 3E), and by 2.25-fold (Fig. 3F) in the Epit muscles. GS phosphorylation was significantly reduced (~60%) only in the Sol of cold-exposed rats (Fig. 3G–I). *Glut4* gene expression was significantly increased by ~8-fold in the Sol (Fig. 4A) of cold-exposed rats, whereas in the EDL (Fig. 4B) and Epit (Fig. 4C) muscles this variable did not differ between control and cold-exposed rats. No significant differences were observed for *Glut1* expression in any of the muscles from cold-exposed rats (Fig. 4A–C). These findings are in line with increased rates of glycogen synthesis and glycogen content that were only observed in the Sol muscles of cold-exposed rats.

### Effects of cold exposure on palmitate oxidation, gene expression of Lpl, Cd36, and Pgc-1α, and AMPK phosphorylation in skeletal muscles

**Figure 5:**
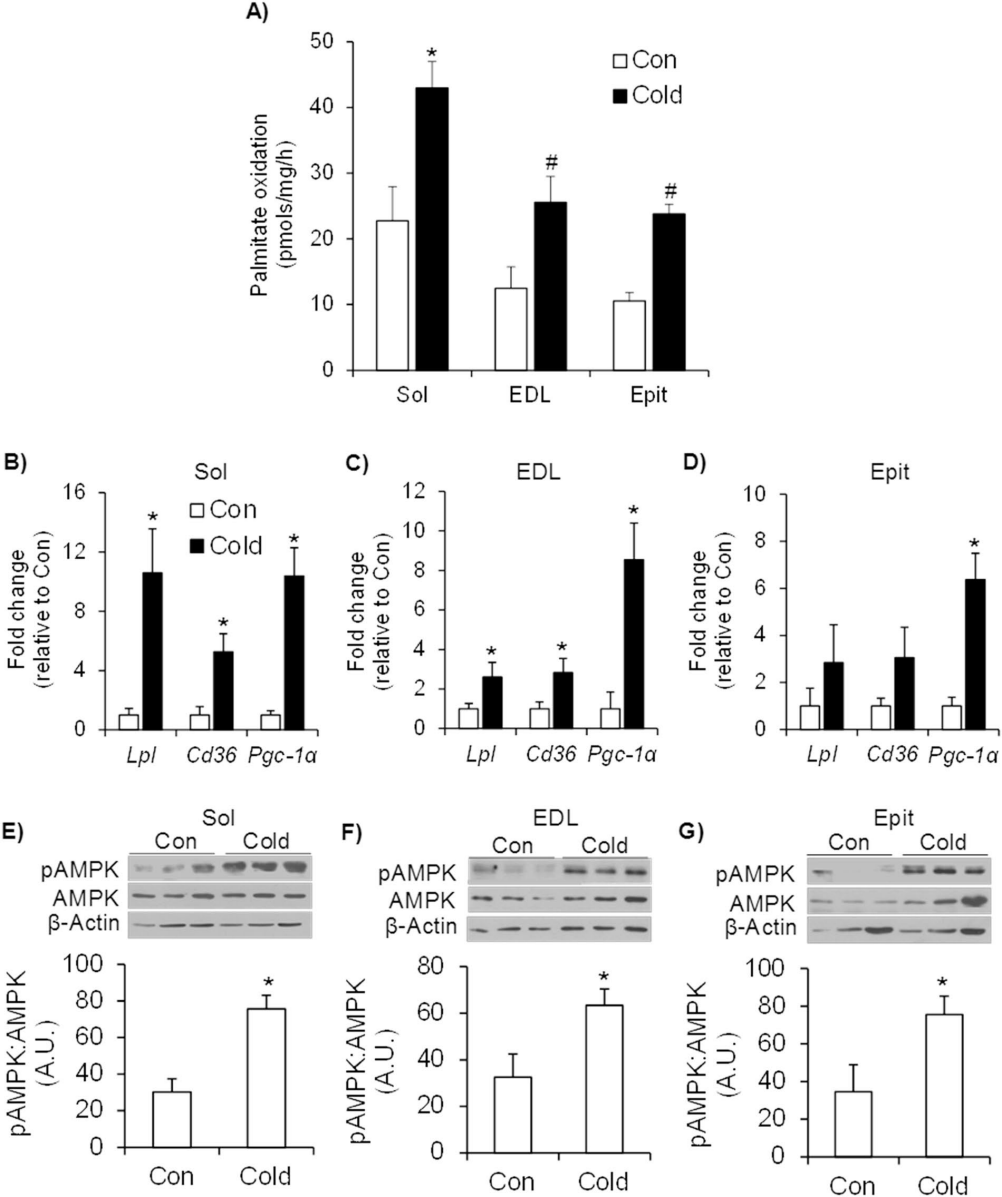
Cold acclimation increases palmitate oxidation (**A**), the expression of genes involved in fatty acid transport and oxidation (**B**–**D**), and the content and phosphorylation of AMPK (**E**–**G**) in the Sol, EDL and Epit muscles. Age- and weight-matched animals were either kept at room temperature or cold-exposed (4 °C) for 7 days. Following the protocol, a strip of each skeletal muscle was incubated in a buffer for measurement of palmitate oxidation, as described in the methods. An additional portion of each skeletal muscle (that was not incubated) was extracted, flash frozen in liquid nitrogen and stored at −80 °C until subsequent western blot or real-time PCR analysis. For A, two-way ANOVA, n = 12. *Indicates interaction between specific muscles and cold exposure (p < 0.05 vs. all other conditions). ^#^Indicates main effect of cold exposure (p < 0.05 vs. respective control (Con)). For B-G, student’s t-test n = 6–9. *p < 0.05 vs. respective control (Con).

Sol muscles exhibited the highest rate of palmitate oxidation (Fig. 5A), but all three muscles significantly increased (~2-fold) (Fig. 5A) their capacity to oxidize palmitate upon cold acclimation. These adaptive responses in FAO were accompanied by significant increases in *Lpl*, *Cd36* and *Pgc-1α* expression in Sol (~11-fold, 5-fold, and 10-fold, respectively) (Fig. 5B) and EDL (~2.6-fold, 2.8-fold, and 8.5-fold, respectively) (Fig. 5C). In the Epit muscle only *Pgc-1α* gene expression was significantly increased (~6.5-fold) by cold exposure, whereas *Lpl* and *Cd36* did not change (Fig. 5D). All alterations in the expression of genes involved in the breakdown and uptake of circulating fatty acids and mitochondria biogenesis were more pronounced in the Sol than EDL and Epit muscles, which is compatible with the higher rates of palmitate oxidation found in Sol than EDL and Epit muscles from cold-exposed rats. AMPK phosphorylation also increased by 2.5-fold in the Sol (Fig. 5E), by 1.95-fold in the EDL (Fig. 5F), and by 2.2-fold in the Epit (Fig. 5G) muscles upon cold acclimation.

### Effects of cold exposure on gene expression of Serca1 and Serca2 and SLN protein content in skeletal muscles

**Figure 6:**
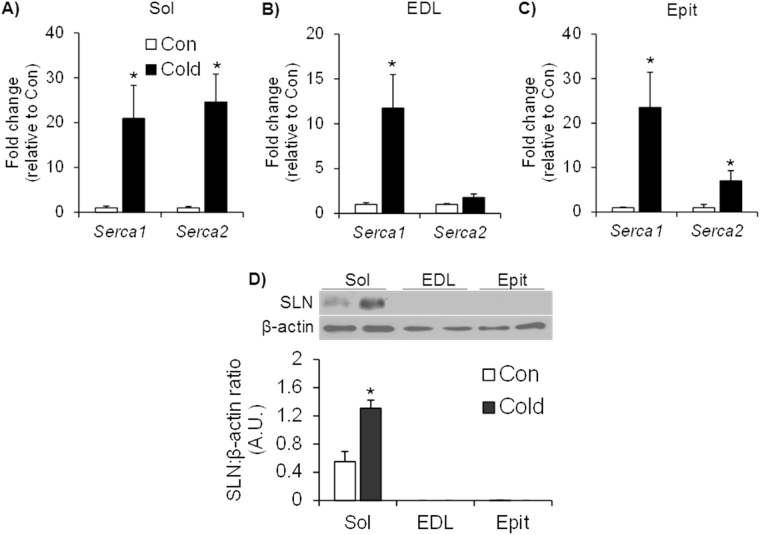
Cold exposure increases the expression of *Serca* in the Sol (**A**), EDL (**B**) and Epit (**C**) muscles but increases the content of SLN only in the Sol (D). Student’s t-test, n = 6–9. *p < 0.05 vs. Control (Con).

Cold increased *Serca1* and *Serca2* gene expressions in the Sol by ~21-fold and 25-fold, respectively (Fig. 6A). In the EDL muscle, cold increased *Serca1* gene expression by ~12-fold, but did not affect the expression of *Serca2* (Fig. 6B). In the Epit muscle, cold increased *Serca1* gene expression by 23.4-fold and *Serca2* by 7-fold (Fig. 6C). SLN was only consistently detected by western blotting in the Sol muscle (Fig. 6D), and upon cold exposure the content of this protein was significantly increased by ~2.4-fold in this muscle (Fig. 6D).

## Discussion

The results of this study provide novel evidence that substrate partitioning is distinctly regulated in oxidative and glycolytic muscles under conditions of cold acclimation. This is supported by our findings that the Sol muscle (rich in type I fibers^29^) increased its rates of glucose oxidation and glycogen synthesis, whereas in the EDL and Epit (rich in type IIb fibers^29^) these variables were not significantly affected either under basal or insulin-stimulated conditions after 7 days of cold exposure. In fact, despite enhancing their ability to utilize glucose to fuel its energy metabolism, Sol muscles increased by ~80% their ability to synthesize glycogen in response to insulin, which was also accompanied by 50% and 30% increases in glycogen content under basal and insulin-stimulated conditions, respectively. Importantly, cold-acclimated rats were hyperphagic, but exhibited reduced insulinemia in comparison to controls. This indicates that cold-induced accumulation of glycogen in Sol muscles *in vivo* was not driven by elevated circulating insulin levels. Heterogeneity in insulin sensitivity and responsiveness among muscles of different fiber compositions has previously been reported^30^ and our findings demonstrate that under conditions of cold-induced thermogenesis such metabolic heterogeneity actually dictates the magnitude of substrate utilization and the intracellular fate of glucose. It is important to note that our results were collected from control animals that were housed at 22 °C, which is below thermoneutrality for a rat (28 °C)^31^. Thus, these control animals were also exposed to some degree of cold stress, which may have attenuated the magnitude of the differences in metabolic responses and regulatory mechanisms we observed following cold acclimation.

Despite eliciting clear fiber type differences with respect to glucose metabolism, all three muscles displayed increased AKT and GSK3 phosphorylation after acclimating to cold. This is consistent with previous reports that cold exposure increases glucose uptake in oxidative and glycolytic muscles^4,12^. However, analysis of GS phosphorylation revealed that this was reduced only in Sol muscle, which is compatible with our observations that basal glycogen content and insulin-stimulated glycogen synthesis were only enhanced in this muscle. Thus, our findings indicate that the regulation of GS phosphorylation and its activity is also fiber-type specific, and that AKT-induced phosphorylation/deactivation of GSK3 does not suffice for cold exposure to enhance glycogen synthesis in glycolytic muscles. However, it is noteworthy that AKT, GSK3 and GS phosphorylation values were obtained from muscle strips that were not incubated in the presence of insulin. This was because under *in vivo* conditions we found that, despite hyperphagia, circulating insulin levels were actually reduced (~35%) throughout the 7-day cold-acclimation period, which suggested that the signaling cascade that regulates glycogen synthesis was distinctly affected in oxidative and glycolytic muscles under conditions of cold-induced reduced insulinemia. This was actually confirmed by our findings obtained from isolated Sol muscles in which glycogen content was increased under basal conditions after cold acclimation, whereas in EDL and Epit muscles glycogen content did not differ between control and cold-acclimated rats. Importantly, even though AKT, GSK3, and GS phosphorylation was compatible with the results of the glycogen synthesis assay, the values cannot be directly compared to the glycogen synthesis data collected under insulin-stimulated conditions. Previous studies have reported that cold acclimation markedly enhanced insulin-stimulated glucose uptake in Sol and EDL muscles *in vivo*, although this response was much higher in the former than the latter muscles^4,32^. Thus, because cold exposure potentiates insulin action in skeletal muscles^4,32^, it could be that incubation of isolated Sol, EDL, and Epit muscles in the presence of insulin could override basal fiber type differences in glycogen metabolism and lead to enhanced glycogen synthesis in all three muscles from cold-acclimated rats. However, additional studies are required to test whether or not this is indeed the case.

Because glucose metabolism in skeletal muscle is largely dependent on its ability to uptake this substrate, we also assessed the expression of *Glut1* and *Glut4* in all three muscles. Sol muscles elicited a robust increase (~8-fold) in *Glut4* expression, whereas EDL and Epit muscles were quite variable and did not display any significant increases in the expression of either glucose transporter after 7 days of cold exposure. Again, this is consistent with much higher rates of glucose metabolism in Sol than in EDL and Epit muscles. However, it is important to consider that, regardless of gene expression, GLUT4 protein abundance and/or translocation could still be enhanced by cold exposure and facilitate glucose uptake in all muscles. In fact, it has recently been demonstrated that cold acclimation in humans is accompanied by increased GLUT4 localization at the sarcolemma^33,34^, which supports cold-induced enhancement of skeletal muscle capacity to uptake glucose, although in these studies no data was provided with respect to potential fiber type differences. In our studies, GLUT4 translocation could also have been triggered by AMPK activation that actually significantly increased in all three muscles upon cold acclimation. Therefore, based on our findings, at least two signaling pathways could be simultaneously engaged to promote GLUT4 translocation in skeletal muscles under cold acclimating conditions: one mediated by AKT and another by AMPK. Importantly, rat skeletal muscles rich in type I muscle fibers have been reported to contain a higher abundance of GLUT4 protein^35^ compared to muscles rich in type II fibers. Furthermore, human type I muscle fibers have been reported to contain a higher abundance of proteins that phosphorylate glucose (Hexokinase II), and synthesize glycogen (GS)^36^ than type II fibers. Additionally, we have found that *Glut4* expression was markedly increased in the Sol muscle, whereas in EDL and Epit muscles only a trend was observed upon cold acclimation. In this context, cold-induced simultaneous activation of the AKT and AMPK could at least partially explain why a much more pronounced effect on glucose metabolism was observed in Sol than in EDL and Epit muscles.

We have also found that palmitate oxidation was significantly increased in all three muscles after cold acclimation. Interestingly, the relative increase in FAO was similar (2- to 2.25-fold) among all three muscles, indicating that, besides glucose, myocytes also enhanced their capacity to utilize fatty acids to fuel cold-induced thermogenesis. This was consistent with significant increases in *Pgc-1α* expression and AMPK phosphorylation in oxidative and glycolytic muscles upon cold acclimation. We have also found significantly elevated expression of *Lpl* and *Cd36* in Sol and EDL muscles, which are crucial for lipolysis of triglycerides from lipoproteins and fatty acid uptake, respectively. The Sol displayed by far the most robust increase in *Lpl* (~10-fold) and *Cd36* (~5-fold) expression followed by the EDL (2.6- and 2.9-fold, respectively), whereas in the Epit muscle only a trend towards an increase in the expression of these proteins was observed after cold acclimation. Though gene expression data does not provide a direct indication of functional capacity, the mRNA data in this study complement and are compatible with the FAO data we obtained from freshly extracted muscle. As expected, the absolute value of FAO for the Sol muscle was much higher (almost double) than the values obtained for the EDL and Epit in control and cold-acclimated rats. It is remarkable that the Sol muscle sustained such high rates of both fatty acid and glucose oxidation under cold acclimating conditions, and yet increased its glycogen content. This could be attributed to the insulin sensitizing effect of cold acclimation that enhanced glucose uptake and promoted allosteric activation of GS. This has been demonstrated to occur through an increased in intracellular glucose-6-phosphate (G6P) levels^37^. In fact, evidence has been provided that allosteric activation of GS is the primary mechanism by which insulin promotes muscle glycogen accumulation *in vivo* ^37^. This is consistent with our observations that Sol muscles from cold-acclimated rats had increased rates of insulin-stimulated glycogen synthesis. Because EDL and Epit muscles did not elicit any significant enhancement in glucose metabolism, it is likely that the G6P availability was not high enough to activate GS and promote glycogen synthesis under cold acclimating conditions in these muscles.

We had originally hypothesized that SLN-induced SERCA uncoupling could contribute to non-shivering thermogenesis in skeletal muscles. It would do so by increasing ATP turnover and leading to acceleration of the citric acid cycle, glycolysis, and β-oxidation, ultimately resulting in an enhancement of glucose and fat oxidation under conditions of cold acclimation. Gene expression analysis revealed that *Serca1* expression was markedly increased in all three muscles, whereas *Serca2* expression was upregulated in Sol and Epit, but not in the EDL of cold-acclimated rats. We also tried to assess SERCA protein levels in all three muscles using a commercially available antibody to further validate our mRNA data, but we could not acquire a clear signal that allowed us to determine whether protein levels were affected by cold acclimation. Thus, despite fiber type differences, our findings suggest that in all muscles SERCA-mediated hydrolysis of ATP to transport Ca^2+^ across the membrane was enhanced to some extent, with potential to contribute to cold-induced thermogenesis in these tissues. However, it has been previously reported that SERCA1a and SERCA2a protein contents are reduced and increased, respectively, in cold-acclimated mice^38^. This indicates that it is the latter isoform of SERCA that seems to play an important role in non-shivering thermogenesis is skeletal muscles. With respect to SLN, we only detect this protein in the Sol muscle, and its content was indeed significantly increased upon cold acclimation in this muscle. Of note, it has been previously demonstrated that SLN protein content is increased by cold acclimation in the red portion of gastrocnemius^38^, which is consistent with our observations that muscles rich in type I fibers have the capacity for SLN-mediated SERCA uncoupling. This is also in line with other reports of muscle fiber-type specific expression of SLN^10,23,24^, and provides evidence that SLN-mediated SERCA uncoupling following cold acclimation may increase the energy requirements of muscles with high content of type I fibers. It is possible that these increased energy demands could be met in the Sol muscles, which showed the highest absolute rates of fatty acid and glucose oxidation following cold acclimation when compared to the EDL and Epit.

In summary, here we show that fiber type composition plays a major role in determining the metabolic fate of glucose and fatty acids in skeletal muscles under conditions of cold-induced thermogenesis. The molecular mechanisms that drive these metabolic adaptive responses to cold in oxidative and glycolytic muscles overlap to some extent and involve the activation of AKT and AMPK. However, only in the highly oxidative Sol muscle GS activity seems to be induced and ultimately leads to glycogen accumulation. The molecular machinery involved in mitochondrial biogenesis and FAO was also upregulated in all muscles and characterized by increased expression of *Pgc-1α, Lpl*, and *Cd36*, although the absolute rate of FAO was much higher in oxidative than glycolytic muscles upon cold acclimation. We have also found that the expression of SERCA isoforms is fiber type-specific and that the capacity for SLN-mediated SERCA uncoupling seems to be present only in muscles rich in type I fibers. Despite these major fiber type-specific metabolic differences, cold acclimation promoted an insulin-sensitizing effect and enhanced the disposal of glucose and fatty acids in skeletal muscles. These metabolic adaptive responses to cold acclimation may be of great importance for diseases characterized by hyperglycemia and dyslipidemia such as obesity and type II diabetes.

## Methods

### Reagents

Fatty acid-free bovine serum albumin (FA-free BSA), palmitic acid, triethanolamine hydrochloride (TRA), amyloglucosidase and hexokinase/glucose-6-phosphate dehydrogenase were obtained from Sigma (St. Louis, MO, USA). [1- ^14^C] palmitic acid was from Perkin Elmer (Woodbridge, ON, Canada). D-[U- ^14^C] glucose was from GE Healthcare (Mississauga, ON, Canada). Protease (cOmplete Ultra Tablets) and phosphatase (PhosSTOP) inhibitors were from Roche Diagnostics GmbH (Mannheim, Germany). Glucose was measured by the glucose oxidase method using a OneTouch Ultra Mini Monitor. The NEFA kit was from Wako (Mountain View, CA, USA) and the rat insulin ELISA kit was from Alpco (Salem, NH, USA). All antibodies were purchased from Cell Signaling (Danvers, MA, USA) except for SLN which was purchased from Millipore (Billerica, MA, USA).

### Animals and cold exposure

Male albino rats (Wistar strain) were age- and weight-matched (~400 g) and allocated to either the control or cold-exposed group. All animals were housed individually on a 12/12 h light/dark cycle and fed standard laboratory chow (Lab Diet Cat #5012) *ad libitum* throughout the 7-day protocol. The control animals were housed at 22 °C, while those exposed to cold were kept at 4 °C. Food intake and body weight measurements were taken for 5 days prior to (baseline) and every day during the cold acclimation period. A blood sample was collected in the fed state each morning at 09:00 am, centrifuged, and the plasma frozen at −80 °C until subsequent analysis. Upon completion of the protocol the animals were anesthetized at approximately 09:00 am using ketamine/xylazine (0.2 ml per 100 g body weight) and the muscles immediately extracted for subsequent analysis. All efforts were made to minimize suffering. The protocol containing all animal procedures described in this study was specifically approved by the Committee on the Ethics of Animal Experiments of York University (York University Animal Care Committee, YUACC, permit number 2016-5) and performed strictly in accordance with the YUACC guidelines.

### Measurement of glycogen synthesis and palmitate and glucose oxidation

Sol, EDL, and epitrochlearis (Epit) muscles were chosen because of their distinct fiber-type distributions, which clearly characterizes them as mainly oxidative or glycolytic. The percentages of type I, type IIa, type IIb and type IIx in Sol, EDL, and Epit muscles are 88/12/0/0, 2/12/57/29, and 8/13/51/28^29^, respectively. Thin strips of each muscle (~20 mg) were mounted onto stainless steel wire clips in order to maintain a relaxed resting length and incubated as previously described^39^. Muscle strips were then incubated in plastic scintillation vials at 37  °C for 1 h in 2 ml of gassed (45 min with O_2_:CO_2_–95:5% vol:vol) Krebs Ringer buffer (0.154 M NaCl, 0.154 M KCl, 0.11 M CaCl_2_, 0.154 M MgSO_4_, 0.154 M KH_2_PO_4_, 0.154 M NaHCO_3_, pH 7.4, with 5.5 mM glucose and 30 mM HEPES (KRBH)) supplemented with 3.5% FA-free BSA. For glycogen synthesis and glucose oxidation muscle strips were incubated in 2 ml of KRBH-3% FA-free BSA with 0.2 μCi/ml of D-[U- ^14^C] glucose, either in the absence or presence of insulin (100 nM)^39^. Subsequently, muscle strips were digested in 0.5 ml of KOH (1M) and an aliquot (400 μl) was used to detect the amount of radiolabeled glucose incorporated into glycogen as previously described^39^. For palmitate oxidation muscle strips were incubated in 2 ml of KRBH-3%BSA with 0.2 μCi/ml of [1- ^14^C] palmitic acid and 200 μM non-labelled palmitate. Oxidation was measured by the production of ^14^CO_2_ either from glucose or palmitate as previously described^40,41^.

### Measurement of glycogen content

Muscle strips were incubated in 2 ml of KRBH-3% FA-free BSA with 0.2 μCi/ml of D-[U- ^14^C] glucose, either in the absence or presence of insulin (100 nM)^39^. At the end of the incubation, the muscles were digested in 1 M KOH and 100 μl of the digested solution was used for the assessment of glycogen content. The pH of the muscle digest was titrated to 4.8 and 500 μl of acetate buffer (pH 4.8) containing 0.5 mg/ml amyloglucosidase was added. Hydrolyzation of the muscle glycogen was allowed to proceed overnight at room temperature. The solution was neutralized prior to adding 1 ml of TRA buffer (TRA 0.3 M, MgSO_4_ 4.05 mM, KOH 1 N, pH 7.5, with hexokinase/glucose-6-phosphate dehydrogenase 250 U/ml, ATP 1 mM and NADP 0.9 mM) for the enzymatic analysis of glucose. The samples were then incubated at room temperature for 30 min and absorbance was read at 340 nM wavelength in a spectrophotometer (Ultraspec 2100 pro; Biochrom Ltd., Cambridge, UK)^39^.

### RNA isolation and quantitative PCR

A portion of each skeletal muscle that did not undergo incubation was flash frozen in liquid nitrogen and stored at −80 °C until RNA isolation. RNA was isolated from skeletal muscle tissue using TRIzol™ (ThermoFisher Scientific, Waltham, MA, USA) and complimentary DNA (cDNA) was made from 2 μg of extracted RNA using the ABM EasyScript™ Reverse Transcriptase cDNA synthesis kit (Diamed, Mississauga, ON, Canada), according to the manufacturer’s instructions. Primers were designed using the software PrimerQuest (IDT) based on probe sequences available at the Affymetrix database (NetAffx™ Analysis Centre, http://www.affymetrix.com/analysis) for each given gene. Real-time PCR analysis was performed using a Bio-Rad CFX96 Real Time PCR Detection System (Bio-Rad, Mississauga, ON, Canada) using the following amplification conditions: 95 °C (10 min); 40 cycles of 95 °C (15 s), 60 °C (60 s). All genes were normalized to the control gene TBP, and relative differences in gene expression between treatment groups were determined using the ΔΔCt method^42^. Values are presented as fold increases relative to the Con group. The primers used in this study are as follows: Glut4 (5′-CAT TCT CGG ACG GTT CCT CAT-3′ (Forward), 5′-CCA AGG CAC CCC GAA GAT-3′ (Reverse)), Glut1 (5′-AAT GAG CTA GGA GGC TTT ACC GCA-3′ (Forward), 5′-TGG AAG AGA CAG GAA TGG GCG AAT-3′ (Reverse)), Lpl (5′-TTG AGA AAG GGC TCT GCC TGA GTT-3′ (Forward), 5′-TGC TTC TCT TGG CTC TGA CCT TGT-3′ (Reverse)), Cd36 (5′-ACG ACT GCA GGT CAA CAT ACT GGT-3′ (Forward), 5′-TGG TCC CAG TCT CAT TTA GCC ACA-3′ (Reverse)), Pgc-1α (5′-ACC GTA AAT CTG CGG GAT GAT GGA-3′ (Forward), 5′-ATT CTC AAG AGC AGC GAA AGC GTC-3′ (Reverse)), Serca1 (5′-TTC ATT GCT CGG AAC TAT CTG G-3′ (Forward), 5′-GGG CTG GTT ACT TCC TTC TTT-3′ (Reverse)), Serca2 (5′-CTG TAG GTC TGA TGG TTC TGT TTA-3′ (Forward), 5′-CTA GGC GAA GGG ACA GAA AC-3′ (Reverse)).

### Western blotting analysis of content and phosphorylation of proteins

A portion of each skeletal muscle that did not undergo incubation was flash frozen in liquid nitrogen and stored at −80 °C until western blotting analysis. Muscle tissues from the Sol, EDL and Epit were homogenized in a buffer containing 25 mM Tris-HCl, 25 mM NaCl (pH 7.4), 1 mM MgCl_2_, 2.7 mM KCl, 1% Triton-X and protease and phosphatase inhibitors (Roche Diagnostics GmbH, Mannheim, Germany). Samples were diluted 1:1 vol:vol with 2x Laemmli sample buffer, heated to 95 °C for 5 min and subjected to SDS-PAGE. The following primary antibodies were used at a dilution of 1:1,000: P-AKT (Ser 473, 60 kDa, Cell Signaling Cat# 9271); AKT (60 kDa, Cell Signaling Cat# 9272); P-GSK3α (Ser 21, 51 kDa, Cell Signaling Cat# 9327); GSK3α (51 kDa, Cell Signaling Cat# 5676); P-GS (Ser 641, 85–90 kDa, Cell Signaling Cat# 3891); GS (84 kDa, Cell Signaling Cat# 3886); P-AMPK (Thr 172, 62 kDa, Cell Signaling Cat# 2535); AMPK (62 kDa, Cell Signaling Cat# 2532); SLN (6 kDa, Millipore Cat# ABT13). β-actin (45 kDa, Cell Signaling Cat# 4967) was used as a loading control. Phospho and total blots were run simultaneously on two separate membranes loaded with the same samples, the same amount of protein and in the same order. Blots for the housekeeping proteins were taken from either the phospho or the total membranes.

### Statistical analyses

Statistical analyses were assessed by unpaired, two-tailed t-test and two-way ANOVA with Bonferroni post-hoc test. Statistical significance was set at p < 0.05.

### Data availability

The datasets generated during and/or analyzed during the current study are available from the corresponding author on reasonable request.
